# Evaluating the population-level effects of overdose prevention sites and supervised consumption sites in British Columbia, Canada: Controlled interrupted time series

**DOI:** 10.1371/journal.pone.0265665

**Published:** 2022-03-22

**Authors:** Dimitra Panagiotoglou

**Affiliations:** Department of Epidemiology, Biostatistics and Occupational Health, McGill University, Montreal, Quebec, Canada; New York City Department of Health and Mental Hygiene, UNITED STATES

## Abstract

**Background:**

On 14 April 2016, British Columbia’s Provincial Medical Health Officer declared the overdose crisis a public health emergency, sanctioning the implementation of new overdose prevention sites (OPS) and supervised consumption sites (SCS) across the province.

**Methods:**

We used the BC Centre for Disease Control’s Provincial Overdose Cohort of all overdose events between 1 January 2015 and 31 December 2017 to evaluate the population-level effects of OPSs and SCSs on acute health service use and mortality. We matched local health areas (LHA) that implemented any site with propensity score matched controls and conducted controlled interrupted time series analysis.

**Results:**

During the study period, twenty-five OPSs and SCSs opened across fourteen of British Columbia’s 89 LHAs. Results from analysis of LHAs with matched controls (i.e. excluding Vancouver DTES) were mixed. Significant declines in reported overdose events, paramedic attendance, and emergency department visits were observed. However, there were no changes to trends in monthly hospitalization or mortality rates. Extensive sensitivity analyses found these results persisted.

**Conclusions:**

We found OPSs and SCSs reduce opioid-related paramedic attendance and emergency department visit rates but no evidence that they reduce local hospitalization or mortality rates.

## Introduction

British Columbia (BC) is Canada’s hardest hit province in the ongoing overdose epidemic, with 1535 resident deaths (32.0 deaths per 100,000 population) in 2018 alone [1]. In response to the almost five-fold increase in the annual number of overdose deaths observed in 2015/16 compared with the period between 1999 and 2012 [2], the Provincial Medical Health Officer declared a public health emergency on 14 April 2016 [3]. This enabled the BC Centre for Disease Control (BC CDC), Ministry of Health, Regional Health Authorities, and BC Coroners Services to expand real-time surveillance, and to sanction new harm reduction services [4].

Beginning in December 2016, multiple overdose prevention sites (OPS) and supervised consumption sites (SCS) opened across the province, including two mobile units in Kelowna and Kamloops [5]. These services complemented the two supervised injection facilities (SIF) that already existed in Vancouver (InSite and the Dr. Peter Centre). Similar to SIFs, OPSs and SCSs provide safe, clean spaces for people to consume pre-obtained drugs under the supervision of staff trained to reverse overdoses, without risk of arrest for drug possession [6]. Unlike SIFs, consumption is not limited to injectables. Both OPSs and SCSs also offer critical overdose prevention education, Take Home Naloxone training and distribution, access to drug use equipment, and safe disposal [6–9]. The primary difference between OPSs and SCSs is that OPSs are temporary sites permitted during public health emergencies, and do not require exemption under section 56.1 of Canada’s Controlled Drugs and Substances Act. Further, SCSs provide access to primary care, housing and social assistance programs while OPSs typically do not. Lastly, OPSs are generally run by trained peer workers while SCSs must always have a nurse on site. It is worth noting that in other contexts (e.g. other provinces, internationally) the distinctions described here may not be salient.

Although evidence demonstrates SIFs, and by extension OPSs and SCSs, help reduce the spread of blood borne infectious diseases (e.g. HIV, hepatitis C) and prevent accidental overdoses and consequent morbidity (e.g. anoxic brain injury, rhabdomyolosis) and mortality, they remain politically controversial and, until recently, cumbersome to implement [10–14]. Some policy makers, residents and business operators continue to vehemently oppose their implementation on moral grounds, and beliefs that these harm reduction interventions: a) encourage drug-related crimes and public consumption, b) condone rather than treat addiction, and c) burden limited health resources [15–17].

Critical gaps in the literature contribute to the underrating of OPSs and SCSs as crucial health services. Much of the evidence is specific to the concentrated drug use epidemics of Vancouver’s Downtown Eastside (DTES) and Sydney’s ‘red light’ district, predates the current opioid overdose epidemic, and is specific to injection drug use [10, 12]. This leaves the effects of OPSs and SCSs unclear in contexts where the population is diffuse (i.e. geographically scattered), services are not restricted to people who inject drugs, mobile rather than fixed sites are offered, and during periods of intervention scale-up [18]. The recent implementation of OPSs and SCSs across a variety of settings in BC and over time presents an excellent natural experiment to evaluate the population-level effects of this harm reduction intervention.

In this study, we compare changes in rates of opioid-related mortality and health service use between communities that implemented any variant of OPS/SCS with communities that did not. We hypothesize that OPSs and SCSs reduce opioid-related mortality and health service use rates.

## Materials and methods

### Study design

This is a controlled interrupted time series study using data from the retrospective BC overdose cohort for the period 1 January 2015 to 31 December 2017.

### Setting

British Columbia is Canada’s most western province and has a population of approximately 4.8 million residents. The province provides single payer coverage of inpatient and outpatient health services through its Medical Services Plan. Residents excluded from the insurance program include newly landed immigrants and people covered under federal insurance programs including refugees, asylum seekers, military personnel and First Nations’ members (representing less than 4% of the population) [19].

In 2012, fentanyl was first detected in the illicit drug supply, and 4% of the province’s 270 overdose deaths were fentanyl-related. By 2019, fentanyl was detected in over 85% of the province’s 984 drug overdose deaths [1]. Here, illicit overdose events include indication of street drugs (controlled and illegal: heroin, cocaine, MDMA, methamphetamine, illicit fentanyl), and medications not prescribed to the decedent but obtained/purchased on the street, from unknown means, or where origin of drug not known.

To test our hypotheses, we compared outcomes at the local health area (LHA) level. LHAs are a mutually exclusive and exhaustive classification of the land area [20]. Although they have no administrative functions, until 2019, LHAs were the smallest geographic boundaries used for health services planning and delivery [21]. There are eighty-nine LHAs ranging from 2,000 residents in sparsely populated remote communities to 485,000 in heavily dense urban centres. BC’s largest city, Vancouver, is comprised of six LHAs ranging from 63,000 to 145,000 residents. During the study period, fourteen LHAs implemented the intervention, and seventy-five did not.

### Study population and data sources

We used the BC Provincial Overdose Cohort [22–26] which captures all coroner confirmed drug-related mortality and non-fatal opioid-related overdose events involving health service provider interactions that occurred in British Columbia. Because the province’s coroner services are required to investigate and determine cause of death for all “unnatural, sudden and unexpected, unexplained, or unattended deaths,” [27] there is a two-year lag between confirmed opioid-related mortality and access to data for research purposes.

The cohort included all events treated with naloxone administered by paramedics; calls to the Drug and Poison Information Centre about an opioid-related event; coroner-determined illegal drug overdose deaths; hospital admissions with ICD-10 code T40.0, T40.1, T40.2, T40.3, T40.4, or T40.6; emergency department visits with ICD-10 code T40.0 or T40.6; and outpatient physician visits with ICD-9 code 965.0 or E850.0. Non-fatal opioid-related overdose events treated with naloxone by a peer in the community or staff at an OPS or SCS were not included *unless* there was follow-up with a health services provider. Although BC Coroners Service data include deaths involving any street drugs (heroin, cocaine, MDMA, methamphetamine, illicit fentanyl etc.), as well as medications not prescribed to the deceased, combinations of the preceding with prescribed medications, and those overdoses where the origin of the drug was not known, we restricted our analysis to deaths involving opioids (heroin, codeine, oxycodone, morphine, hydromorphine, methadone, fentanyl and analogues, etc.).

For individuals with multiple opioid-related overdose events during the study period, each event was included separately in the data set. Event location was assigned as the LHA where the event initial occurred or where the deceased was found if no previous contact with health services was made. Detailed description of the cohort is available elsewhere [22].

### Outcomes

The primary outcome was coroner confirmed mortality. Secondary outcomes were opioid-related health service encounters defined as hospitalizations using admissions records in the Discharge Abstract Data base, emergency department visits captured in the National Ambulatory Care Reporting System, and paramedic attendance in BC Emergency Health Services data holdings.

### Data preparation and analysis

We estimated the population-level effects of the newly implemented OPS/SCSs using propensity score matched interrupted time-series analysis. This quasi-experimental study design enabled us to estimate the effects of the intervention despite the lack of randomization by geography [28].

For each LHA that implemented the intervention we selected a matched counterfactual from the pool of LHAs that did not begin operating any OPS or SCS within twelve months of the exposed LHA’s implementation month to account for confounding by indication. Owing to constraints with the data, we worked with LHA characteristics plausibly predicting the implementation of OPS or SCS (i.e. population-level age, sex, and income demographics and opioid-related overdose mortality rate). Low income residents were those covered by PharmaCare Plan C–the provincial drug plan available only to individuals and families receiving income assistance. We applied matching with replacement using a maximum-likelihood logit model to calculate each LHA’s propensity score [29]. The matching algorithm used these scores and a caliper of width equal to 0.15 of the standard deviation of the logit of the propensity score to create two evenly matched groups while accounting for the variance-bias trade-off [30].

For each outcome, we organized exposed-control LHA pairs by study time and created twelve weighted monthly outcome rates per 100,000 population by exposure group pre- and post-implementation, censoring on the month of implementation. For LHA-level population size, we used Statistics Canada Census data (periods 2011, 2016) and linear interpolation and extrapolation to estimate monthly values during the study period. Where LHAs implemented more than one OPS or SCS during the study period (e.g. Vancouver’s Downtown Eastside, Victoria, and Surrey), we set study time = 0 as the operation month of the first new OPS and considered subsequent OPS/SCS openings within the LHA as ‘scale-up’. As part of our sensitivity analysis, we assigned the LHA of the first point of medical contact (e.g. hospital) where opioid-related overdose event location was missing.

We inspected time trends for autocorrelation using the 2-sided Durbin-Watson test and visual plots of the autocorrelation and partial autocorrelation functions [31, 32], adjusting where necessary. We tested our hypotheses using ordinary least squares and segmented regression, by fitting the following regression model, per outcome:

$${\text{Outcome}}_{\text{jkt}}=\beta_{0}+\beta_{1}{\text{time}}_{\text{t}}+\beta_{2}{\text{group}}_{\text{k}}+\beta_{3}{\text{group}}_{\text{k}}{\text{time}}_{\text{t}}+\beta_{4}{\text{level}}_{\text{jt}}+\beta_{5}{\text{trend}}_{\text{jt}}+\beta_{6}{\text{level}}_{\text{jt}}{\text{group}}_{\text{k}}+\beta_{7}{\text{trend}}_{\text{jt}}{\text{group}}_{\text{k}}+\varepsilon_{\text{jkt}}$$

where *j* was the intervention, *t* was the study time in monthly intervals pre- (negative time) and post-intervention (positive time), and *k* distinguished between intervention and control group. Significant values for coefficients β_6_ and β_7_ indicate an effect of the intervention on exposed LHA after controlling for level and trend changes in the controls, respectively. Where a matched control could not be found for an exposed LHA, we analyzed this LHA separately using a traditional interrupted time series analysis. Data were prepared using SAS 9.4 [33]. All statistical analyses and graphics were conducted in R 3.6.1 [34] using the *nlme* [35], *car* [36] and *ggplot2* [37] packages.

McGill University’s Institutional Review Board (Certificate Number: A12-E79-18A) and the University of British Columbia’s Research Ethics Board (Certificate Number: H18-03361) approved this study. This study only uses secondary data and did not require consent from participants included in the datasets.

## Results

Between January 2015 and December 2017, there were 36,576 unique overdose events in British Columbia. Of these, 26,223 (71.7%) were attended by paramedics, 24,171 (66.1%) included a visit to an emergency department, 3356 (9.2%) events resulted in a hospitalization, and 3604 (9.9%) resulted in mortality (not mutually exclusive events). Of the 36,576 overdose events, 5836 (16.0%) were missing the event location. The majority (76.3%) of events missing location information were first captured in emergency department (ED) visit records, and are likely indicative of patients who self-transported to the ED. When we restricted our analysis to overdose events with complete location information, we were left with 30,736 (84.0% of the 36,576) opioid-related overdose events, 26,220 (100.0% of the 26,220) attended by paramedics, 19,550 (80.9% of the 24,171) emergency department visits, 2370 (70.6% of the 3356) hospital admissions, and 3526 (97.9% of the 3604) overdose mortalities.

During the study period, fourteen LHAs implemented at least one OPS–some of which have since transitioned to SCSs (Table 1). When we matched with replacement, we found eleven control LHAs for thirteen exposed LHAs (Tables 1 and 2). Propensity score matching did not identify a suitable control for Vancouver’s DTES and we did not include data from this LHA as part of the aggregate analyses.

**Table 1:** Implementation month-year and location of overdose prevention site or supervised consumption site, with propensity score matched local health area.

Site Name	Type	Weekly booth-hours per 100,000 population§	Exposed LHA	Access restrictions	Implementation Month	Control LHA	Source
Downtown \ Rutland*	mOPS	38.91	23 –Central Okanagan	None	April 2017	166 –South Vancouver	[5, 38]
ASK Wellness \ Crossroads Housing*	mOPS	66.49	24 –Kamloops	None	June 2017	40 –New Westminster	[5]
Positive Living Fraser Valley	OPS	112.07	34 –Abbotsford	Shelter based, available to residents only	December 2016	43 –Coquitlam	[39]
Gateway of Hope	OPS	109.99	35 –Langley	Shelter based, available to residents and guests	December 2016	44 –North Vancouver	
Salvation Army Ridge Meadows Ministry Shelter	OPS	313.81	42 –Maple Ridge	Shelter based, available to residents only	December 2016	22 –Vernon	
Maple Ridge Temporary Homeless Shelter	OPS				December 2016		
Anita Place	OPS				May 2017		
Prince George AIDS Prevention Program	OPS	38.50	57 –Prince George	None	December 2016	4 –Windermere	[40]
Johnson Street Community*	OPS	304.27	61 –Greater Victoria	Shelter based,	December 2016	20 –Salmon Arm	[41, 42]
				available to			
				residents only			
Our Place Society	OPS			None	December 2016		
Victoria Cool Aid Society	OPS			None	February 2017		
AIDS Vancouver Island–Victoria	OPS			None	March 2017		
Cowichan Valley	OPS	269.39	65 –Cowichan Valley	None	September 2017	22 –Vernon	[43, 44]
Canadian Mental Health Association	OPS	121.38	68 –Nanaimo	None	February 2017	165 –Midtown	[45]
Port Alberni Shelter Society	OPS	340.87	70 –Alberni	None	May 2017	41 –Burnaby	[43]
AIDS Vancouver Island–Comox Valley	OPS	28.30	71 –Courtenay	None	March 2017	46 –Sunshine Coast	[43]
AIDS Vancouver Island	OPS	41.83	72 –Campbell River	None	May 2017	7 –Nelson	
Maple Hotel	OPS	1467.23	162 –Downtown Eastside	None	December 2016	**Unmatched**	[46–48]
VANDU	OPS			None	December 2016		
Overdose Prevention Society	OPS			None	December 2016		
SisterSpace	OPS			Women only	May 2017		
Powell Street Getaway	SCS			None	July 2017		
Molson Site and Lab	OPS			None	September 2017		
Lookout Society–City Parkway*	OPS	83.19	201 –Surrey	Shelter based,	December 2016	43 –Coquitlam	[49, 50]
				available to	May 2017		
				residents only	May 2017		
Quibble Creek Sobering & Assessment Centre*	OPS			None	July2017		
SafePoint*	OPS			None			
Lookout Society–Whalley Boulevard	OPS			None			

§Booth-hours include only hours provided by first OPS/SCSs opened in LHA and are calculated as the number of spaces multiplied by the number of hours per 100,000 population as estimated for the month of initial operation. Where more than one OPS/SCS was opened in the first month all hours across these sites was included. For Vancouver’s Downtown Eastside, booth-hours exclude pre-existing InSite and Peter Wall Centre.

*Since first opening, some overdose prevention sites (OPSs) have transitioned to supervised consumption sites (SCSs); others have expanded access from residents/guests only to general public

mOPS = mobile overdose prevention site.

**Table 2:** Characteristics of exposed and control LHAs in aggregate and restricted to propensity-score matched cohorts, 2015.

	Aggregate Cohort				Propensity-Score Matched Cohort			
LHA Characteristics	Exposed	Unexposed	Difference	P-Value	Exposed	Unexposed	Difference	P-Value
No (%)	14 (16.5)	71 (83.5)			13 (54.2)	11 (45.8)		
PharmaCare Plan C (%, SD)	7.13 (1.56)	5.82 (2.16)	-1.31 (-2.88, 0.27)	0.0998	8.69 (6.56)	5.81 (2.47)	-2.88 (-6.39, 0.63)	0.1001
Among opioid-naïve deaths (%, SD):								
Males	52.03 (3.84)	51.17 (3.90)	-0.65 (-4.15, 2.42)	0.5903	53.13 (5.52)	53.59 (7.93)	0.45 (-3.96, 4.88)	0.8370
Age (mean, SD)	77.46 (2.35)	79.32 (1.42)	1.85 (0.17, 3.54)	0.0325	77.11 (2.61)	76.11 (4.69)	-1.00 (-2.82, 0.82)	0.2700
Mortality Rate per 100,000 (mean, SD)	790.30 (155.3)	722.60 (215.3)	-67.74 (-224.90, 89.46)	0.3812	786.30 (150.0)	852.20 (382.3)	65.93 (-55.54, 187.4)	0.2811
Among opioid related deaths (%, SD):								
Males	74.08 (11.20)	76.24 (16.30)	2.16 (-9.53, 13.84)	0.7055	74.69 (10.99)	68.36 (30.89)	-6.32 (-16.47, 3.81)	0.2169
Age (mean, SD)	57.16 (10.36)	59.50 (7.38)	2.35 (-5.41, 10.10)	0.5365	57.08 (9.96)	62.12 (12.68)	5.04 (-2.35, 12.43)	0.1774
Mortality Rate per 100,000 (mean, SD)	88.31 (24.37)	64.23 (27.43)	-24.08 (-46.34, -1.82)	0.0354	23.44 (22.1)	13.34 (11.56)	-10.10 (-18.10, -2.10)	0.1174

Focusing on the year before and after implementation in exposed and matched control LHAs captured 19,119 opioid-related overdose events (14,141 in exposed, and 4,978 in control LHAs) across the thirteen exposed LHAs (n~1,859,477 residents) and eleven matched LHAs (n~1,203,260 residents). Of these unique overdose events, 16,551 were attended by paramedics (12,277 in exposed, and 4,274 in control); 13,569 included a visit to the emergency department (10,704 in exposed, and 2,865 in control); 1,760 included a hospitalization (1,273 in exposed, and 487 in control); and 2,129 resulted in death (1,499 in exposed, and 630 in control).

**Fig 1:**
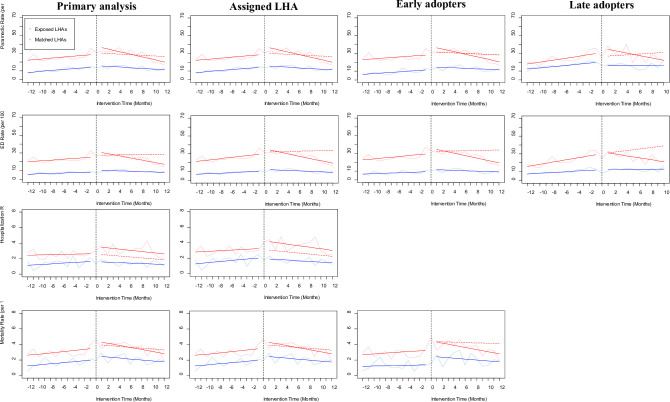
Panel of propensity score matched interrupted time series results, by analysis. Solid lines indicate observed trends, dashed are predicted.

After accounting for observed changes in levels and trends in matched controls, our analysis found no significant changes in monthly overdose mortality or hospitalization rates. However, we did observe significant decreases in trends of paramedic attended event rates (1.14 fewer events per 100,000 population per month; 95% CI: -1.99 to -0.28) after an initial (level) increase of 7.43 events per 100,000 population (95% CI: 1.27 to 13.58) immediately following implementation (Table 3 and Fig 1). In other words, paramedic events increased 30.1% after implementation of OPS/SCS but declined 3.0% per month thereafter, for an absolute difference of 6.19 fewer paramedic attended events per 100,000 (23.5% relative decrease) by twelve months post-implementation compared with expected. Similarly, we found emergency department visits declined (1.25 fewer events per 100,000 population; 95% CI: -1.95 to -0.55) after an increase of 3.93 events per 100,000 population (95% CI: -1.14 to 9.00) post-implementation, for a 22.8% initial increase followed by a 3.6% decline per month ultimately resulting in 11.11 fewer emergency department visits per 100,000 (39.0% relative decrease) than expected at twelve months post-implementation.

**Table 3:** Interrupted time series for exposed and propensity score matched controls. All output is reported per 100,000 population.

	All matched LHAs		Assigned LHAs		Early adopters		Late adopters	
	β (95% CI)	P-value	β (95% CI)	P-value	β (95% CI)	P-value	β (95% CI)	P-value
**Paramedic Attendance**	16,551 attended events: 12,277 in exposed, and 4,274 in controls		No missing event location		13,277 attended events: 10,512 in exposed, and 2,715 in controls		3,324 attended events: 1,765 in exposed, and 1,559 in controls	
Matched control, pre-implementation (β_0_)	14.67 (11.85, 17.49)	<0.001	14.67 (11.85, 17.49)	<0.001	11.90 (8.64, 15.16)	<0.001	20.34 (16.21, 24.48)	<0.001
Matched controls’ trend, pre-implementation (β_1_)	0.52 (0.12, 0.92)	0.0143	0.52 (0.12, 0.92)	0.0143	0.45 (-0.00, 0.91)	0.0603	0.66 (0.07, 1.24)	0.0336
Difference between exposed and matched controls, pre-implementation (β_2_)	14.35 (10.36, 18.34)	<0.001	14.35 (10.36, 18.34)	<0.001	16.74 (12.14, 21.35)	<0.001	10.41 (4.56, 16.26)	0.0012
Difference in trend between exposed and matched controls, pre-implementation (β_3_)	0.04 (-0.52, 0.61)	0.8817	0.04 (-0.52, 0.61)	0.8817	0.00 (-0.65, 0.65)	0.9916	0.41 (-0.42, 1.24)	0.3399
Matched controls, post-implementation (β_4_)	1.33 (-3.02, 5.69)	0.5512	1.33 (-3.02, 5.69)	0.5512	2.99 (-2.03, 8.02)	0.2494	-4.17 (-10.96, 2.63)	0.2370
Matched controls’ trend, post-implementation (β_5_)	-0.89 (-1.49, -0.29)	0.0058	-0.89 (-1.49, -0.29)	0.0058	-0.73 (-1.42, -0.03)	0.0468	-0.62 (-1.67, 0.43)	0.2549
Difference between exposed and matched controls, post-implementation (β_6_)	7.43 (1.27, 13.58)	0.0227	7.43 (1.27, 13.58)	0.0227	6.32 (-0.79, 13.42)	0.0888	9.27 (-0.35, 18.88)	0.0665
Difference in trend between exposed and matched controls (β_7_)	-1.14 (-1.99, -0.28)	0.0123	-1.14 (-1.99, -0.28)	0.0123	-1.18 (-2.16, -0.20)	0.0234	-1.82 (-3.30, -0.34)	0.0210
**Emergency Department Visits**	13,569 ED visits: 10,704 in exposed, and 2,865 in controls		15,320 ED visits: 12,018 in exposed, and 3,302 in controls		12,625 ED visits: 10,360 in exposed, and 2,265 in controls		2,695 ED visits: 1,658 in exposed, and 1,037 in controls	
Matched control, pre-implementation (β_0_)	8.77 (6.44, 11.09)	<0.001	10.53 (8.18, 12.88)	<0.001	9.63 (6.83, 12.44)	<0.001	12.38 (9.04, 15.71)	<0.001
Matched controls’ trend, pre-implementation (β_1_)	0.22 (-0.11, 0.55)	0.2027	0.29 (-0.04, 0.62)	0.0921	0.24 (-0.16, 0.63)	0.2465	0.40 (-0.07, 0.87)	0.1026
Difference between exposed and matched controls, pre-implementation (β_2_)	17.05 (13.76, 20.34)	<0.001	19.61 (16.29, 22.93)	<0.001	20.45 (16.48, 24.42)	<0.001	18.05 (13.33, 22.76)	<0.001
Difference in trend between exposed and matched controls, pre-implementation (β_3_)	0.26 (-0.21, 0.72)	0.2863	0.42 (-0.05, 0.89)	0.0852	0.37 (-0.19, 0.93)	0.2020	0.81 (0.14, 1.47)	0.0230
Matched controls, post-implementation (β_4_)	1.97 (-1.62, 5.56)	0.2877	1.89 (-1.73, 5.51)	0.3111	2.28 (-2.05, 6.60)	0.3082	-0.27 (-5.74, 5.21)	0.9248
Matched controls’ trend, post-implementation (β_5_)	-0.42 (-0.91, 0.08)	0.1082	-0.56 (-1.06, -0.06)	0.0324	-0.47 (-1.07, 0.12)	0.1275	-0.33 (-1.17, 0.52)	0.4498
Difference between exposed and matched controls, post-implementation (β_6_)	3.93 (-1.14, 9.00)	0.1366	3.60 (-1.52, 8.72)	0.1753	3.76 (-2.36, 9.88)	0.2358	2.16 (-5.58, 9.91)	0.5874
Difference in trend between exposed and matched controls (β_7_)	-1.25 (-1.95, -0.55)	0.0011	-1.51 (-2.21, -0.80)	<0.001	-1.51 (-2.36, -0.66)	0.0011	-1.99 (-3.18, -0.80)	0.0023
**Hospital Admissions**	1,760 hospitalizations: 1,273 in exposed, and 487 in controls		2,082 hospitalizations: 1,504 in exposed, and in 578 controls		Results censored: cell sizes smaller than 5 encounters for some periods.			
Matched control, pre-implementation (β_0_)	1.69 (1.17, 2.22)	<0.001	2.11 (1.54, 2.69)	<0.001				
Matched controls’ trend, pre-implementation (β_1_)	0.04 (-0.03, 0.12)	0.2453	0.07 (-0.01, 0.15)	0.1078				
Difference between exposed and matched controls, pre-implementation (β_2_)	0.96 (0.22, 1.71)	0.0147	1.17 (0.35, 1.98)	0.0075				
Difference in trend between exposed and matched controls, pre-implementation (β_3_)	-0.03 (-0.13, 0.08)	0.5984	-0.03 (-0.14, 0.08)	0.6339				
Matched controls, post-implementation (β_4_)	-0.06 (-0.87, 0.75)	0.8799	-0.16 (-1.05, 0.73)	0.7304				
Matched controls’ trend, post-implementation (β_5_)	-0.08 (-0.19, 0.04)	0.1902	-0.11 (-0.23, 0.01)	0.0792				
Difference between exposed and matched controls, post-implementation (β_6_)	0.97 (-0.18, 2.11)	0.1052	1.12 (-0.14, 2.38)	0.0878				
Difference in trend between exposed and matched controls (β_7_)	-0.02 (-0.18, 0.14)	0.8258	-0.03 (-0.20, 0.14)	0.7449				
**Overdose Mortalities**	2,129 deaths: 1,499 in exposed, and 630 in controls		No missing event location		1,669 deaths: 1,259 in exposed, and 410 in controls		Results censored: cell sizes smaller than 5 encounters for some periods.	
Matched control, pre-implementation (β_0_)	2.03 (1.48, 2.57)	<0.001	2.03 (1.48, 2.57)	<0.001	1.37 (0.69, 2.06)	<0.001		
Matched controls’ trend, pre-implementation (β_1_)	0.07 (-0.01, 0.14)	0.0986	0.07 (-0.01, 0.14)	0.0986	0.01 (-0.08, 0.11)	0.7698		
Difference between exposed and matched controls, pre-implementation (β_2_)	1.47 (0.70, 2.24)	<0.001	1.47 (0.70, 2.24)	<0.001	1.91 (0.94, 2.88)	<0.001		
Difference in trend between exposed and matched controls, pre-implementation (β_3_)	0.01 (-0.10, 0.12)	0.8878	0.01 (-0.10, 0.12)	0.8878	0.04 (-0.10, 0.17)	0.6144		
Matched controls, post-implementation (β_4_)	0.48 (-0.36, 1.32)	0.2712	0.48 (-0.36, 1.32)	0.2712	1.09 (0.03, 2.15)	0.0499		
Matched controls’ trend, post-implementation (β_5_)	-0.13 (-0.25, -0.01)	0.0329	-0.13 (-0.25, -0.01)	0.0329	-0.07 (-0.22, 0.07)	0.3356		
Difference between exposed and matched controls, post-implementation (β_6_)	0.44 (-0.75, 1.63)	0.4731	0.44 (-0.75, 1.63)	0.4731	0.06 (-1.44, 1.56)	0.9362		
Difference in trend between exposed and matched controls (β_7_)	-0.08 (-0.23, 0.09)	0.3622	-0.08 (-0.23, 0.09)	0.3622	-0.11 (-0.32, 0.09)	0.2846		

Our analysis of Vancouver’s DTES using a traditional ITS with no control found significant declines in trends of opioid-related overdose mortality (-2.76, 95% CI: -4.39 to -1.31), paramedic attendance (-36.43, 95% CI: -52.90 to -19.95), emergency department visits (-26.31, 95% CI: -39.29 to -13.32), and hospitalization (-2.12, 95% CI: -4.16 to -0.08) rates per 100,000 population per month.

To test the robustness of the results we ran a series of sensitivity analyses. For previously excluded events with missing location information, we assigned the location of the first point of medical contact. Doing so captured 34,256 (93.7%) opioid-related overdose events, 26,220 (100.0%) of which were attended by paramedics, 23,266 (96.3%) included emergency department visits, 3217 (95.6%) included hospitalization, and 3528 (97.9%) resulted in death. Restricting analysis to twelve periods before and after implementation, we had 20,960 opioid-related overdose events (+9.6%; 16,701 in early adopters–LHA that implemented OPS/SCS commencing December 2016 and matched controls, and 4,259 in late exposed–LHA that implemented their first site after December 2016 and matched controls); no change in the number of paramedic attended events (13,277 early and 3,324 late adopter matched pairs); 15,320 emergency department visits (+12.9%; 12,625 early and 1,658 late adopter matched pairs); 2,082 hospitalizations (+18.3%); and no change in deaths (1,669 early and 460 late adopter matched pairs). When we repeated our analysis using assigned event location, and again separating early adopters and their controls, and late adopters and their controls, the results remained unchanged (Table 3 and Fig 1).

## Discussion

The results from our analyses were mixed. We found no statistically significant changes in opioid-related overdose mortality and hospitalization rates’ levels or trends following OPS/SCS implementation across matched LHA pairs. However, we did observe significant declines in monthly rates of paramedic attendance and emergency department visits following the introduction of OPS/SCSs. In the case of paramedic attended events, the overall decline in trend was preceded by an initial spike in level of events post-implementation. The observed level change was not surprising given original guidelines for OPSs developed with the BC CDC and The Portland Hotel Society recommended calling emergency health services and transferring care to paramedics for all overdose events reversed with naloxone [51–53]. With time, protocols for when to call emergency health services changed and allowed more discretion by the attending peer or staff (e.g. Fraser Health’s 2018 Policy and Protocol Recommendations for Service Providers included text on determining a priori with clients when to call 9-1-1) [54].

Our results echo findings reported in other evaluations of OPS/SCSs locally and internationally. A 2007 Australian report by the National Centre for HIV Epidemiology and Clinical Research for the New South Wales Department of Health found “no statistically significant differences in the rates of decrease of opioid-related deaths between Kings Cross [where the SCS was implemented] and the rest of [New South Wales]” but did observe a significant decrease in ambulance attendances during the six years of follow-up [55]. Similarly, a report by British Columbia’s Island Health Authority examining the early effects of recently implemented OPSs found some effect on local ambulance dispatches and emergency department visits but did not examine effects on mortality [43]. Finally, a mathematical model of the individual and combined impacts of BC’s recently implemented or otherwise expanded harm reduction interventions (i.e. Take Home Naloxone program, OPSs, and opioid agonist treatment) estimated that 5% (95% credible interval (CRI) = 3–7%) of deaths were averted by OPSs alone, with 1.3 (95% CRI = 0.9–1.7) deaths averted per site per month between April 2016 and December 2017 [56]. The model’s estimates may appear at odds with the reported 3476 overdose events reversed by OPS/SCSs in the first year post-implementation [13, 56, 57]. However, although overdoses account for substantial morbidity, not all events are fatal in the absence of medical or peer intervention (e.g. naloxone or oxygen); and some clients experience multiple overdose events [55, 58–61].

In a separate set of analyses examining the effects of newly implemented OPS/SCSs in Vancouver’s DTES using traditional interrupted time series without a matched control, the positive effects observed therein may be a result of intervention-specific and community-level features unique to this LHA. For example, higher volume facilities, longer hours of operation, and no client restrictions (Table 1) may overcome barriers to access. Further, OPS/SCSs in the DTES may benefit from a legacy of strong, grass roots activism that reduce drug use-related stigma, thereby improving acceptability of this intervention locally. However, without an appropriate control, we cannot dismiss the possibility that regression to the mean or some uncontrolled historical bias explain the observed effect.

Over 69% of people who died from an illicit drug overdose in British Columbia between 2016 and 2017 consumed alone at the time of the fatal event [1]. This population is plausibly different from the target population of OPS/SCSs including Vancouver’s Insite whose goal was to “attract the [individuals] who were at highest risk of health-related harms and those responsible for public order problems (e.g., public injection drug use)” [5, 62]. Persistent stigma and police presence may impede the social acceptability and uptake of OPSs by at-risk and vulnerable populations [63]. Meanwhile, hours of operation, facility capacity (see Table 1), residence requirements and absence of safe inhalation rooms limit the effectiveness of these services [43, 64]. Together, these factors may explain why there was limited uptake in some communities (e.g. 13 visits daily at Duncan site) [44] and no observed effect on overdose mortality rates.

Our study had several limitations. For starters, to ensure the privacy of individuals captured in the cohort, the smallest geographic unit available for analysis was the LHA. This restricted our ability to test the effects of OPSs at a more granular level, particular given the distance-decay effect observed in Marshall et al.’s 2011 seminal paper. This work demonstrated that Vancouver’s Insite had a significant effect on overdose mortality within 500m of the supervised injection facility but minimal impact outside this radius [14]. More recently, the Island Health Authority’s evaluation of OPSs in Campbell River, Courtenay, Cowichan Valley, and Port Alberni found their effects on paramedic dispatches waned outside a 1km radius [43]. While these studies suggest our unit of analysis was not fine enough to isolate truly localized effects on overdose mortality and hospitalization rates, they also indicate that it is unlikely population mobility across LHA boundaries diluted the effects of the new OPS/SCSs. Importantly, Marshall et al.’s work was specific to the very concentrated DTES community, aggregated over five years of pre- and post-data, and did not explore changes in monthly rates. Conversely, our work covered geographically dispersed clients, evaluated the effects of the intervention in the year following implementation, and examined changes in outcomes by level (immediate) and trends (over time). To replicate Marshall et al.’s analysis across our exposed regions while protecting individual’s privacy, accounting for temporal trends, and correcting for autoregression, we would need a much longer observation period with study intervals of quarters or years instead of months–negatively impacting the timeliness and relevance of the findings [28, 65].

With respect to the propensity score matching approach applied, we were confined to using individual-level administrative data to create population-level summary health statistics and had to assume that these, combined with LHA-level 2015 overdose mortality rates, sufficiently predicted the probability of treatment assignment. This approach may not adequately capture the qualitative differences in local political leadership and culture which foster the successful implementation of harm reduction interventions, as well as differences in illicit drug market toxicity (all unobservable) which affects event counts. In other words, the LHAs that implemented SCSs may differ from the pool of potential controls such that residual confounding biases the effect estimates. This may be less of a problem for OPSs. These historically unsanctioned, nimble, grass-roots, and peer-initiated responses to the neglected needs of people who use illicit substances generally precede local political will and support; with several implemented during the observation period despite local back lash [66, 67]. Given most sites began as OPSs, the concern that treated LHAs were sufficiently different from controls with respect to local support and thereby probability of treatment initiation, while non-negligible, should not diminish our findings. Meanwhile, BC Coroners Services’ publicly available data of fentanyl-related and overall illicit overdose deaths, show consistent trends of overdose mortalities where fentanyl, W-18 and carfentanil were detected in treated vs. control regions [68–70]. The observed outcome rate trends pre-implementation (see β_3_ estimates in Table 3 for paramedic attendance, hospitalization and mortality rates) met the parallel trends assumption critical in many econometric study designs corroborating the suitability of exposed-control pairings and further mitigating concerns that unobservable differences in political leadership, culture or illicit drug supply should bias our findings. Finally, segmented regression can compensate for imperfect controls (see β_3_ estimates in Table 3 for ED visits) in ways that other study designs, such as difference-in-differences, cannot [71]. That said, local differences in the acceptability of the intervention may have facilitated or impeded use by at-risk individuals, affecting their observed effectiveness.

The Overdose Cohort does not include events that occurred at OPS/SCSs unless additional health services were sought. One interpretation of the results then may be that the effects of OPS/SCSs on paramedic attended events and ED visits is due to a substitution effect–these harm reduction sites reversed overdose events that would otherwise have utilized health services; but did not necessarily reduce the number of overdose events. Results from the BC CDC’s publicly reported Overdose Response Indicators [72] combined with reports published by the health authorities support this interpretation of the decline in overdose events. For example, between December 2016 and November 2017 there were 235,466 visits and 1,253 overdoses reversed in the newly implemented OPS/SCSs (excluding Vancouver’s Insite with 119,395 visits and 1432 overdoses reversed) [43, 73].

Further, we were unable to directly account for other factors that may have influenced the rates of overdose mortality and health services used. Our data did not allow us to control for the time-varying distribution of Take Home Naloxone (THN) kits, potency of drugs on the illicit market, or changes in access to opioid agonist treatment. However, publicly available Overdose Response Indicators produced by the BC CDC and the Ministry of Mental Health and Addictions suggest no corresponding changes to the number of THN kits distributed, or clients dispensed opioid agonist treatment that better account for the changes in health service use and overdose mortality rates observed post-implementation [57]. Meanwhile, BC Coroners Services’ data suggest fentanyl contamination trends (both relative and absolute) were more aligned between propensity score matched treated-control pairs than between treated and neighboring geographic units, helping rule out that differences in street drug toxicity explain the lack of effect observed for hospitalizations and mortalities post-implementation [68–70].

Lastly, we were unable to find a suitable control for Vancouver’s DTES given the LHA’s unique population profile and needs. At the time of intervention implementation, the DTES’ outcome rates were at least one order of magnitude greater than those observed in any other LHA (deaths = 50.86; hospitalizations = 66.07; emergency department visits = 578.33; and paramedic attendance = 664.24 per 100,000 population) with unparalleled growth in rates. Similarly, creating a synthetic control using Abadie et al’s. method was infeasible, since the outcomes fell outside the convex hull of available controls [74]. As such, we interpret these findings separately and with caution.

Despite these limitations, this is the largest evaluation of the population-level effects of OPS/SCSs across a variety of settings, available to date. We explored the impact of the intervention on opioid-related overdose mortality and health service use using the most comprehensive data set available, across a variety of settings (i.e. rural, remote and urban communities), and over time. Using matched controls and applying a multiple baseline approach allowed us to better account for potential historical bias [75]. Our analyses included approximately two thirds of BC’s population, and over 80% of opioid-related overdose events that occurred in the year before and after implementation of OPS/SCSs in matched pair LHAs. Overall, our analyses showed new OPS/SCSs reduced health service use substantially but we were unable to detect an effect on overdose mortality rate a year after implementation. To our knowledge, this is the first study to comprehensively explore the effects of OPS/SCSs in contexts where the target population is not concentrated in a small geographic area.

## Conclusion

The overdose epidemic in BC is unique to that observed elsewhere [76]. In the United States, the epidemic is described as a triple wave of overdose deaths starting with prescription opioids, followed by heroin, and more recently, fentanyl [77, 78]. Other regions in Canada demonstrate a similar epidemiological transition from prescription opioids to illicit substances. Despite the early epidemiological differences, regions across North America are now contending with similar illicit opioid overdose epidemics.

In Canada, OPSs and SCSs are increasingly employed as a strategy to reduce overdose-related morbidities and mortality. Our study shows that this harm reduction intervention reduces paramedic and emergency department use; and the results may be of particular interest to jurisdictions considering mobile units or implementation in less dense communities (e.g. the Appalachian region of the United States). Additional research is needed to understand the effects of operating hours, service volume, residence requirements and police presence. Studies quantifying the impacts of SCSs as low barrier access to other health services for marginalized populations may identify additional benefits.

## List of abbreviations

BC: British Columbia
BC CDC: British Columbia Centre for Disease Control
CrI: Credible interval
DTES: Downtown Eastside
ED: Emergency department
HCV: Hepatitis C virus
HIV: Human immunodeficiency virus
ITSA: Interrupted time series analysis
LHA: Local health area
OPS: Overdose prevention site
SCS: Supervised consumption site
SIF: Supervised injection facility
THN: Take Home Naloxone
